# The effect of electrical stimulation on cortical cells in 3D nanofibrous scaffolds

**DOI:** 10.1039/c8ra01323c

**Published:** 2018-03-20

**Authors:** Qinwei Xu, Lin Jin, Cheng Li, Shreyas Kuddannayai, Yilei Zhang

**Affiliations:** School of Mechanical & Aerospace Engineering, Nanyang Technological University 50 Nanyang Avenue Singapore 639798 Singapore ylzhang@ntu.edu.sg +65-6792-4062 +65-6790-5953; Henan Provincial People's Hospital Number 7 Weiwu road Zhengzhou 450003 P. R. China jinlin-1982@126.com; Henan Key Laboratory of Rare Earth Functional Materials, Zhoukou Normal University 466001 P. R. China; Singapore Centre for Environmental Life Sciences Engineering, Interdisciplinary Graduate School, Nanyang Technological University 60 Nanyang Drive Singapore 637551 Singapore

## Abstract

Cellular behaviors are significantly affected by cellular microenvironment, including mechanical supports, electrical and chemical cues, *etc.* Three dimensional conductive nanofibers (3D-CNFs) provide the capability to regulate cellular behaviors using mechanical, geometrical and electrical cues together, which are especially important in neural tissue engineering. However, very few studies were conducted to address combined effects of 3D nanofibrous scaffolds and electrical stimulation (ES) on cortical cell cultures. In the present study, polypyrrole (PPy)-coated electrospun polyacrylonitrile (PAN) nanofibers with a 3D structure were successfully prepared for the cortical cell culture, which was compared to cells cultured in the 2D-CNFs meshes, as well as that in the bare PAN nanofibers, both in 2D and 3D. While smooth PAN 3D nanofibers showed dispersive cell distribution, PPy coated 3D-CNFs showed clusters of cortical cells. The combined effects of 3D conductive nanofibers and ES on neurons and glial cells were studied. Different from previous observations on 2D substrates, pulsed electrical stimulations could prevent formation of cell clusters if applied at the beginning of culture, but could not disperse the clusters of cortical cells already formed. Furthermore, the electrical stimulations improved the proliferation of glial cells and accelerate neuron maturation. This study enriched the growing body of evidence for using electrical stimulation and 3D conductive nanofibers to control the culture of cortical cells, which have broad applications in neural engineering, such as implantation, biofunctional *in vitro* model, *etc.*

## Abstract 2

Cellular behaviors are significantly affected by cellular microenvironment, including mechanical supports, electrical and chemical cues, *etc.*

## Graphical abstract

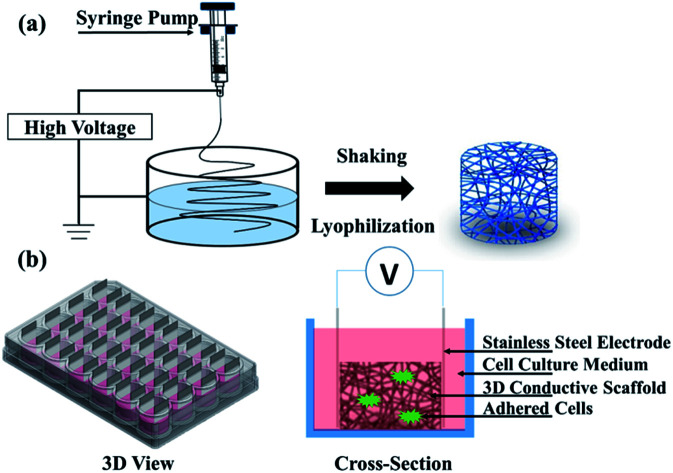

## 1. Introduction

Regeneration capability of adult nervous system is very limited and existing treatments of injuries in central nervous system (CNS) and peripheral nervous system (PNS) are restricted by many factors, like immunological rejections, potential disease transfer, and inhibitory environment formed after injuries.^1–3^ One alternative approach widely studied recently is to fabricate 3D polymeric scaffolds with cells in order to generate tissues suitable for implantation.^4,5^

For neural tissues, multiple cues, such as chemical cues, geometrical cues, and electrical cues, are always presented simultaneously; thus, a platform that could combine different stimuli or cues is highly desired. Nonetheless, many previous studies focused only on separating effects of geometrical cues or electrical stimulations. For example, geometrical cues provided by nanofibers have been studied for neuron growth, which proved that nanofibers could mimic the extracellular matrix (ECM) well in terms of their unique characteristics, including ultrafine continuous fibers, high surface-to-volume ratio, high porosity and so on.^6–9^ In addition, geometrical cues have also been applied to guide stem cells to differentiate into neural lineage,^10,11^ direct orientation of neurons or glials,^10,12–16^ influence proliferation of stem cells,^17^ enhance the neurite outgrowth^18–20^ and guide migration of glial cells.^13,21^ Recently, conductive nanofibers are emerging as one of the most important tools in neural tissue engineering due to the geometrical cues they provide for cell growth and functional expression, as well as the possibility of inducing external electrical stimulations for regulation of cellular behaviors.^22–25^ For example, many studies showed enhanced neurite growth and neuron development caused by electrical stimulations and geometrical cues in 2D conductive nanofibers.^5,14,16,26,27^ One big limitation of 2D nanofibers is that they only support 2D cell culture, *i.e.*, cells could not penetrate and grow inside the fibers. However, 3D cell culture could mimic the complex of *in vivo* conditions, *i.e.*, cells could behave in a manner that is closer to their *in vivo* behaviors. For example, compared to 2D culture, the signaling and gene expression of cells cultured in 3D were closer to those of cells found *in vivo*. With these advantages, a more functional platform could be built for tissue engineering.^28^

In this report, a 3D nanofibrous scaffold was electrospun using polyacrylonitrile (PAN), which was then coated with polypyrrole (PPy) to introduce good biocompatibility and conductivity into the PAN nanofibers while maintain the 3D porous structures.^5,16,29^ Observations confirmed that cells could grow inside the 3D conductive nanofibers (3D-CNFs). The combined effects of the 3D nanofibrous structure and the PPy-coating on cortical cell growth were evaluated in terms of neuron morphology. Furthermore, we reported the influence of electrical stimulation on the morphologies and proliferation of cortical cells and the maturation rate of neurons cultured in these 3D-CNFs.

## 2. Experimental section

### 2.1 Fabrication of PAN nanofibers and conductive nanofibers

**Fig. 1:**
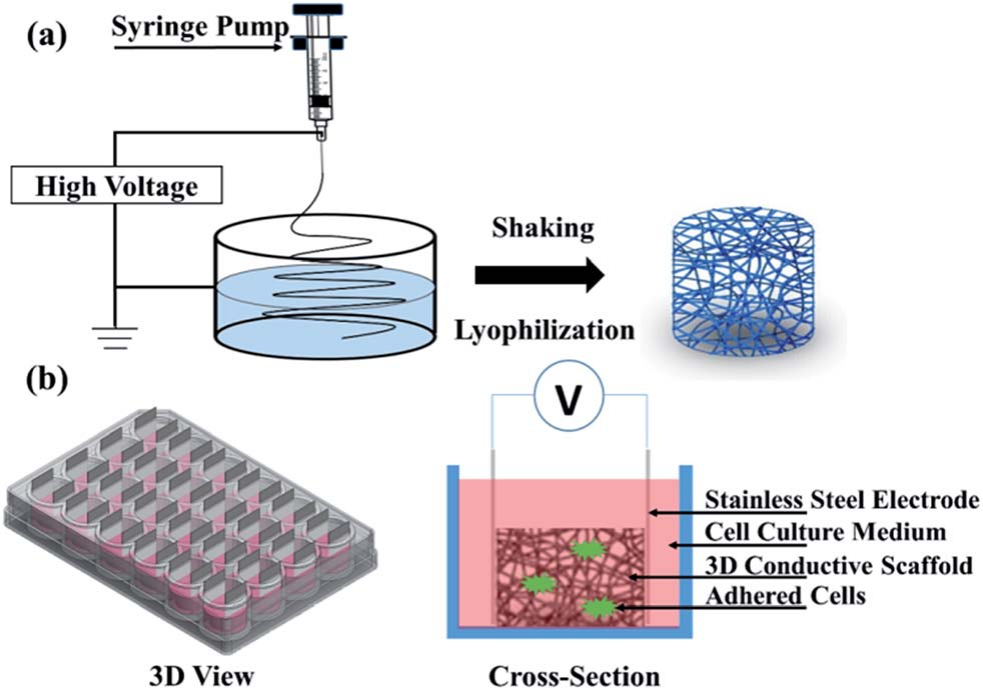
Schematic of (a) electrospinning setup and (b) a bioreactor for electrical stimulation (3D view and cross-section).

Polyacrylonitrile (PAN) is frequently used in biomedical applications such as drug delivery, wound dressing, implantation, and dialysis membrane.^30,31^ Its nanofibers, having diameters in the range from 100 nm to a few microns, could be easily produced *via* the electrospinning technology,^32,33^ which has been used in tissue engineering as scaffolds^34–36^ including 3D scaffolds.^31^ The polypyrrole (PPy) coating was chosen because of its good biocompatibility and high electrical conductivity,^16,37–39^ which could also improve mechanical properties of PAN nanofibers^40^ due to the core–shell structure. Although PPy is considered non-biodegradable and might remain in tissue for a relatively long period, its biocompatibility *in vitro* and *in vivo* has been demonstrated. Within a long period of half year, only light inflammation could be found after a PPy-coated silicone tube was implanted to bridge the gap of rat sciatic nerve.^39^ The fabrication process of scaffolds followed previous work^41^ as illustrated in Fig. 1a. Briefly, the PAN (*M*_n_ = 90 000) was dissolved in *N*,*N*-dimethylformamide (DMF) at a concentration of 10 wt%. Then the polymer solution was constantly stirred until it became homogenous. The electrospinning process was conducted with a customized system, where the solution was fed into a syringe that was capped with a needle (22 gauge, blunt) and pushed by a syringe pump at a rate of 1.0 mL h^−1^. A continuous jetting stream was generated by applying a voltage of 15 kV. The distance between the syringe tip and the collector was 10 cm. 2D PAN nanofibers were deposited onto an aluminium foil-covered collector. For the 3D nanofibers, the PAN nanofibers were spun into the container filled with ethanol solution and the container was shaken every 5 min to let the nanofibers extend fully for the 3D architecture. The 3D PAN nanofibers were then obtained by freeze drying method after washing in deionization (DI) water. The porosity of the 3D nanofibers could be easily changed by dispersing different number of nanofibers into the certain volume of DI water. For our cell culture, the final porosity of all the 3D scaffolds was controlled by dispersing the 3D nanofibers into DI water at a density of 0.125 mg mL^−1^.

The conductive PPy shell layer was coated on the surface of the nanofibers by *in situ* polymerization of pyrrole within FeCl_3_ as the oxidant. Briefly, the nanofibers were immersed in a 0.04 M pyrrole aqueous solution. The polymerization of pyrrole and deposition of PPy were initiated by adding the same volume of 0.084 M FeCl_3_ aqueous solution at room temperature. The mixture was treated by an ultrasonic cleaner for 1 h. Then, the 3D conductive nanofibers (3D-CNFs) were washed by chloroform and freezing dried.

### 2.2 Cell isolation and cell culture

All animal work was approved by the Nanyang Technological University Institutional Animal Care and Use Committee and abided by the Guidelines on the Care and Use of Animals for Scientific Purposes as set out by the National Advisory Committee for Laboratory Animal Research. Cortical tissue was isolated from the cerebral cortex region of the postnatal 1 day old rat pups (P1) as described in the previous work.^42,43^ The tissue was dissociated by trituration after digestion with 20 U mL^−1^ papain (Life Technologies, Singapore) in a dissection buffer adjusted to neutral pH and allowed to digest until a smooth homogeneous precipitate was formed. The cells were then suspended in the culture medium consisting of 50% minimal essential medium (MEM) supplemented with 10% fetal bovine serum and 50% neurobasal medium supplemented with B27 and 0.5 mM glutamine, 25 μM glutamate (Life Technologies, Singapore).

The 3D scaffolds and 2D meshes were fitted into 24-well plates and sterilized by soaking in 70% ethanol solution for 12 hours, followed by washing 3 times with Dulbecco's phosphate buffered saline (DPBS). The samples were sterilized under UV for 2 hours. Before seeding cells, samples were incubated with the culture medium at 37 °C for half an hour. After removing the medium, 100 μL medium with a concentration of 5 × 10^6^ cells per mL were added to the surfaces of samples. The cell-laden samples were incubated at 37 °C with 5% CO_2_ for 30 min to allow cells to attach. After that, 900 μL fresh culture medium was added to the wells. The cell-laden samples were stored at 37 °C with 5% CO_2_ for 24 hours. Then the 24-well plate was shaken gently, and the unattached and dead cells were removed by removing the old medium entirely through pipetting followed by adding the fresh and pre-warmed medium. After that, half old culture medium was changed with pre-warmed fresh medium twice per week.

### 2.3 Electrical stimulation

Electrical stimulation of cell culture was performed on the 3D-CNFs with a self-made bioreactor as showed in Fig. 1b. Isolated cells were seeded into 3D-CNFs at 5 × 10^5^ cells per well. After 24 h in culture, stainless steel electrodes were inserted to form the electrical contact with the 3D scaffolds. A 100 Hz pulsed electrical field of 100 mV cm^−1^ was applied across the two electrodes for 4 h in the incubator (at 37 °C with 5% CO_2_) with a function generator (AFG3022C, Tektronix, USA) daily for one week. Cells were analysed 24 h after electrical stimulation.

### 2.4 Immunocytochemistry and SEM imaging

To assess the cell culture after 7 days, different antibodies were utilized. Anti-Microtubule-Associated Protein-2 (MAP2) (Millipore, Singapore) and anti-Tau (Abcam, Singapore) were chosen to stain the neuron body and axon respectively. Glial fibrillary acidic protein (GFAP) (anti-GFAP antibody, Millipore, Singapore), which is expressed by numerous cell types of the CNS including astrocytes and ependymal cells,^44,45^ were utilized to stain glial cells.

Cell-laden samples were rinsed with 1× PBS and fixed in 10% neutral buffered formalin solution (Sigma, Singapore) for half an hour. Cells were then treated with 0.5% Triton X-100 solution (Sigma, Singapore) for 15 min at room temperature and followed by 45 min incubation in blocking buffer solution consisting 3% bovine serum albumin (BSA) (Sigma, Singapore) and 0.5% fetal bovine serum (FBS) (Life Technologies, Singapore) to avoid non-specific binding of antibodies. The cells were incubated with neuron-specific anti-Microtubule-Associated Protein-2 (MAP2) (Millipore, Singapore) and anti-Tau (Abcam, Singapore) antibodies in 1 : 500 dilution at 4 °C for overnight. On the following day, the cells were exposed to the Alexa Fluor® 488 and Alexa Fluor® 555 (Life Technologies, Singapore) in 1 : 700 dilution at 4 °C for overnight. Finally, cell nuclei were stained with 1 : 1000 diluted DAPI (Life Technologies, Singapore) for 1 min at room temperature.

Synaptophysin antibody (Life Technological, Singapore), as a pre-synaptic biomarker, and post-synaptic density protein 95 (PSD95) (Millipore, Singapore), as a post-synaptic biomarker, were utilized to characterize the synapse. Cells fixed and treated with Triton X-100 as mentioned before were incubated with synaptophysin and PSD95 in 1 : 1000 dilution at 4 °C for overnight. On the following day, the cells were exposed to the Alexa Fluor® 488 and Alexa Fluor® 555 (Life Technologies, Singapore) in 1 : 700 dilution at room temperature for one hour. Finally, cell nuclei were stained with 1 : 1000 diluted DAPI (Life Technologies, Singapore) for 1 min at room temperature.

Fluorescent images from the stained samples were acquired using an inverted confocal laser scanning microscope (Zeiss LSM780). Scaffolds with fixed cells were then washed with DI water and lyophilized for SEM imaging.

### 2.5 Proliferation and maturation

The proliferation of cortical cells was characterized by PrestoBlue cell viability kit (Life Technologies, Singapore). Briefly, the 3D-CNFs with cell culture were washed and incubated in medium containing 10% PrestoBlue reagent for 1 h in a humidified atmosphere at 37 °C and 5% CO_2_. Culture medium containing 10% PrestoBlue reagent was incubated with no cells and served as the blank control. The absorbance of the reduced PrestoBlue reagent was measured at 570 nm while 600 nm was utilized as the reference with a Multiskan Spectrum microplate reader (Thermo Scientific, Singapore). The absorbance reading was utilized to represent the cell amount as the manufacturer's protocol.

The maturation was described as the ratio of the number of mature neurons to the total number of neurons. Cells with MAP2 expression (green fluorescence) were counted as neurons. Among them, the cells with tau expression (red fluorescence) were counted as mature neurons. The cell number was counted with Imaris (Bitplane, USA) by analyzing the fluorescent images.

### 2.6 Statistical analysis

Statistical analysis of proliferation and maturation was performed using two-way Analysis of Variables (ANOVA) followed by Tukey's *post hoc* tests. When *p* < 0.05, results were considered as significant and indicated with *. All experiments were done in triplicate unless otherwise stated. Data in this part were presented in term of mean ± standard deviation (*n* = 3).

## 3. Results and discussion

### 3.1 Characterization of scaffolds

**Fig. 2:**
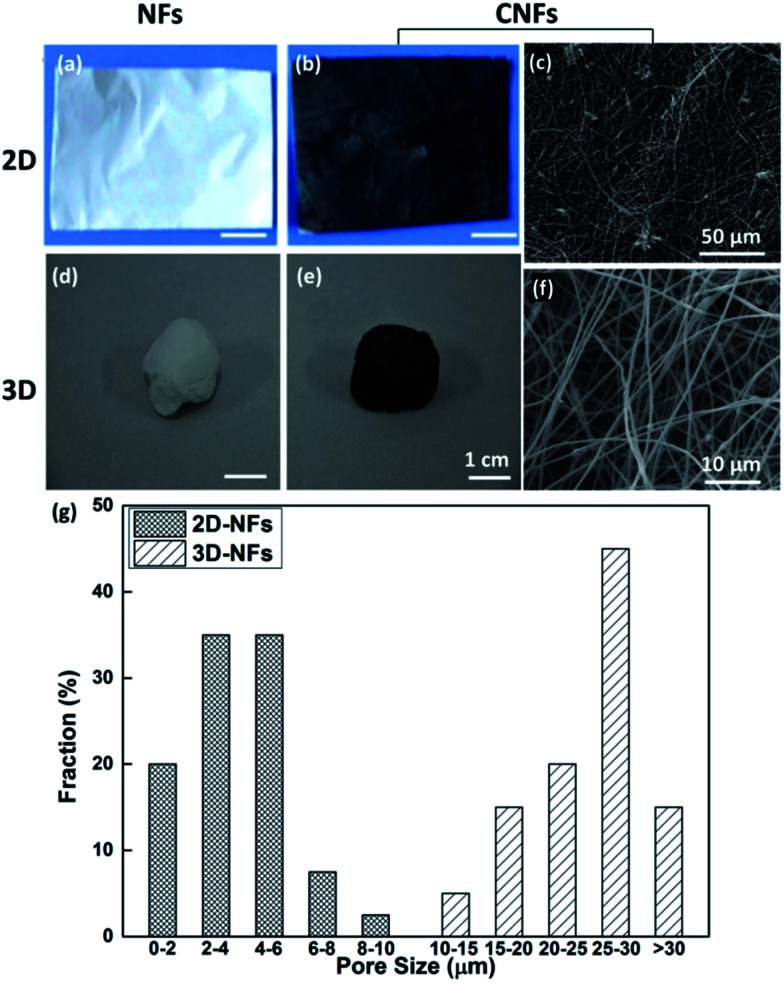
Photographs and SEM images of 2D-NFs mat (a), 2D-CNFs (b and c), 3D-NFs (d), and 3D-CNFs scaffold (e and f). The pore size distribution of 2D-NFs and 3D-NFs (g). (a) and (b) were adapted with permission from ref. 47, Copyright (2016) American Chemical Society.

The optical images of fabricated nanofibers (NFs) and conductive nanofibers (CNFs) as well as the SEM images of 2D- and 3D-CNFs were showed in Fig. 2. The 2D samples were like a thin film (Fig. 2a and b) while the 3D samples had clear 3D architectures (Fig. 2d and e). While the nanofibers in 2D-CNFs exhibited a close-packed structure (Fig. 2c), the 3D-CNFs scaffold had an obvious 3D nanofibrous architecture (Fig. 2f), *i.e.*, nanofibers dispersed in a 3D space without collapsing onto a single layer. The 2D mats have a smaller pore size from 0–10 μm while the 3D-nanofibers have a pore size of 10–30 μm (Fig. 2g), which produced enough spaces to allow cells to penetrate and grow inside the 3D scaffold. The pore size of PAN nanofibers was maintained after polypyrrole coating (not shown here). This ECM-like 3D environment is known to affect cellular responses and functions,^46–49^ which would be further discussed in the following sections.

### 3.2 Cell culture in CNFs and PAN nanofibers

**Fig. 3:**
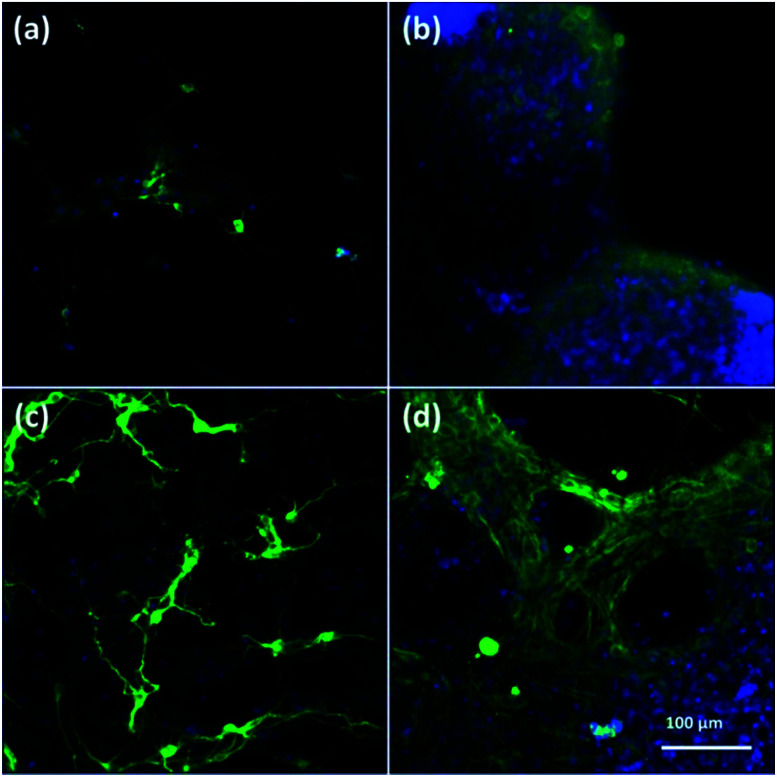
Fluorescent images of anti-MAP2-stained neurons in (a) 2D-PANs, (b) 2D-CNF nanofibers, (c) 3D-PAN and (d) 3D-CNF nanofibers. Green: MAP2; blue: DAPI.

To assess cell culture in CNFs and NFs, fluorescent images of cells were shown in Fig. 3. Firstly, the neuron-specific fluorescent images stained with anti-MAP2 antibody (green) demonstrated the existence of neurons in the PAN and PPy-coated nanofibers. In all kinds of nanofibers, cells are able to form extended neurites and connect with each other, which are essential for cell signal transmission.^50^ However, we observed most of neurons cultured in both 2D (Fig. 3a) and 3D PAN nanofibers (Fig. 3c) tended to grow individually with a sparse distribution, *i.e.*, few neuron clusters in these nonconductive PAN nanofibers. On the contrary, most of neurons cultured in 2D-CNFs (Fig. 3b) and 3D-CNFs (Fig. 3d) tended to grow together to form cell clusters.

**Fig. 4:**
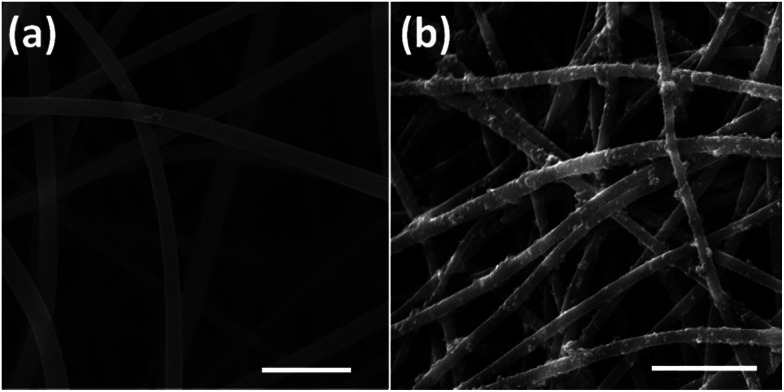
Surface roughness of PAN (a) and PPy-coated PAN nanofibers (b). Scale bar is 5 μm.

This difference of clustering in PAN and PPy-coated nanofibers could be seen in both 2D and 3D samples, which may be attributed to the conductive PPy coating. Previously, it was reported that PPy-coated 2D substrate improved the formation of cell clusters and led to a higher neuron density compared to uncoated substrate.^51^ One possible reason of cluster increase was attributed to the roughness increase in PAN nanofibers after PPy coating because neurons could be negatively affected by surface roughness,^51^ which is true for our 3D samples, *i.e.*, comparing with the smooth surface of PAN nanofibers (Fig. 4a), the PPy-coated nanofibers (Fig. 4b) is much rougher.

### 3.3 Cell culture in 3D

**Fig. 5:**
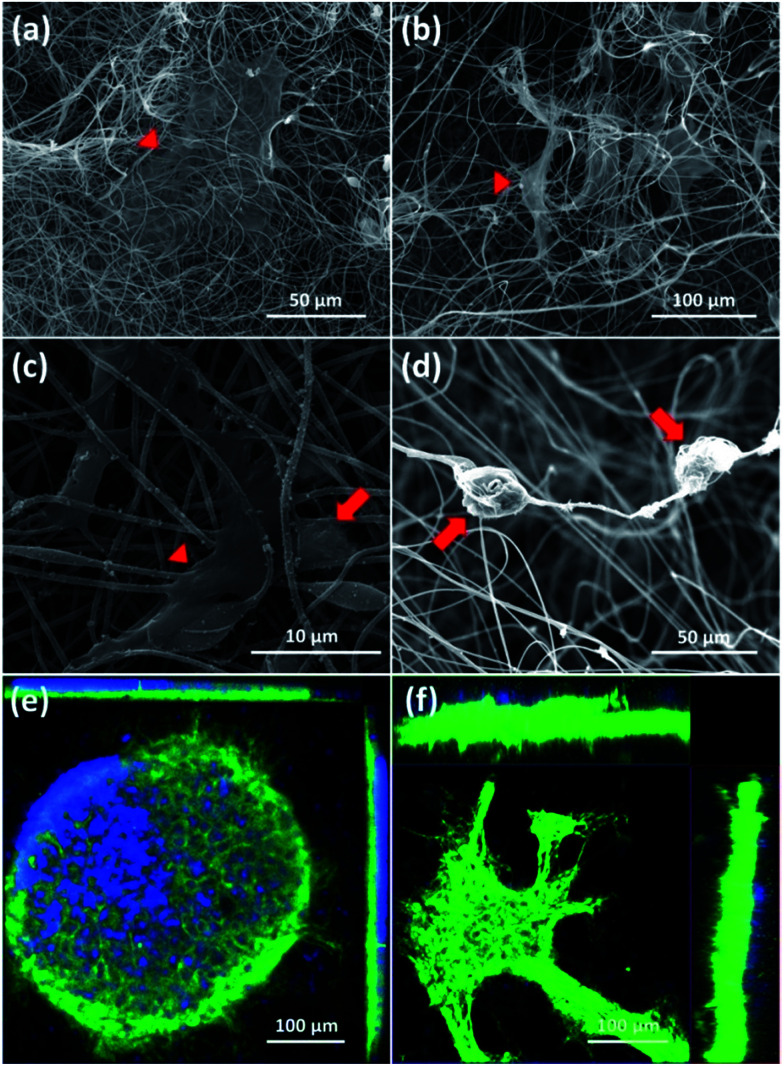
SEM of cortical cells cultured in 2D-CNFs (a and c), 3D-CNFs (b and d) and confocal microscopic ortho-images of cell culture in 2D-CNFs (e) and 3D-CNFs (f). The red arrows and triangles indicated two different cell morphologies respectively. Green: MAP2; blue: DAPI.

First, SEM images of cortical cells cultured onto the surface of 2D-CNFs (Fig. 5a) and in the interior of the 3D-CNFs scaffolds (Fig. 5b) after 7 day *in vitro* culture were shown. The flat cells (indicated by red triangles) were probably glial cells since neurons usually have a round soma. As showed in Fig. 5c, cortical cells in 2D-CNFs stay flat on top of the nanofiber mesh because the dense packing of nanofibers would not allow cells to penetrate and grow inside. In contrast, cortical cells could penetrate and grow in the interior of 3D-CNFs and adhere roundly along the fibers as shown in Fig. 5d.

Furthermore, the 3D confocal microscopic ortho-images confirmed the 2D neuronal network formation on the 2D-CNFs (Fig. 5e) and the 3D neuronal network formation in the 3D-CNFs (Fig. 5f). An even larger depth of the 3D neuronal network could be expected because only part of the whole neuronal network was captured in the view field of the confocal microscopy. Hence, we confirmed that the 3D-CNFs could help neurons and glial cells migrate into the scaffolds thus formed 3D neuronal networks closer to those found *in vivo*. Thus, the 3D-CNFs were selected to study the combined effects of nanofibrous structure and electrical stimulation on cortical cells.

### 3.4 Effect of ES on cell morphology

**Fig. 6:**
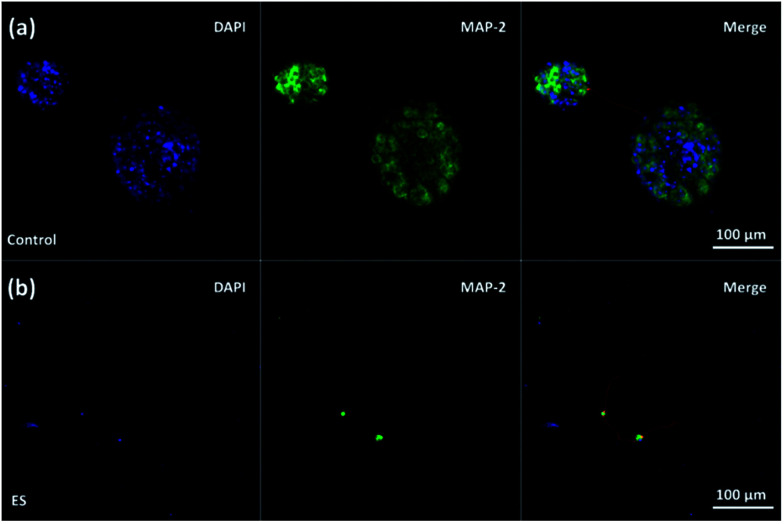
Confocal fluorescent images of neuron morphology after 7 day culture in the 3D-CNFs (a) without and (b) with electrical stimulation. Neurite connections between individual neurons and connection between clusters were marked with red lines.

To study the influence of electrical stimulations, cortical cells were cultured in 3D-CNFs for 7 days with daily pulsed electrical stimulation (ES). As mentioned in the Experimental section, a 100 Hz pulsed electrical field of 100 mV cm^−1^ was applied across the two electrodes for 4 h daily. In non-ES cultures, most neurons aggregated together to form clusters; and neurites (indicated by red line) were found to connect those isolated clusters (Fig. 6a). Unlike the unstimulated 3D-CNFs cultures, ES altered the morphologies of cells, *i.e.*, no cell clusters were found in the electrically stimulated cultures (Fig. 6b). Instead, the electrically stimulated neurons tended to grow individually with neurite connections, which were marked with red lines (in Fig. 6 merge) using Simple Neurite Tracer.^52^

**Fig. 7:**
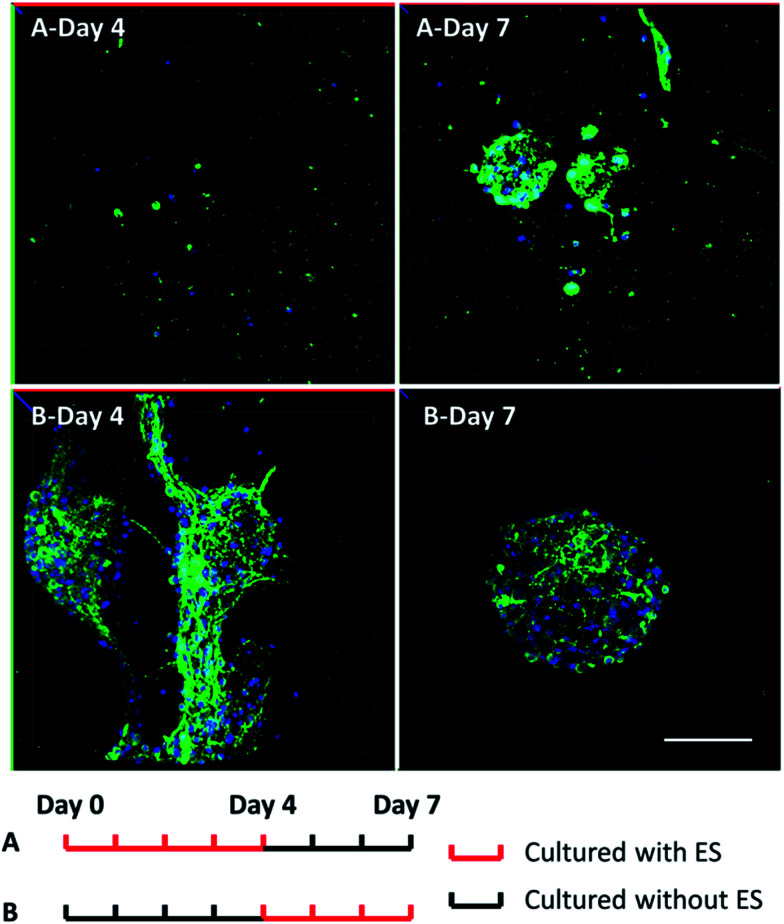
Confocal fluorescent images of neuron morphology after 7 day culture in the 3D-CNFs with different electrical stimulation patterns. Green: MAP2; blue: DAPI. Scale bar: 100 μm.

To further understand whether the clustering behavior change was a short-term or long-term effect, two groups of 3D-CNFs were applied different ES patterns as shown in Fig. 7. Group A was first cultured with ES from Day 0 to Day 4; then cultured without ES till Day 7. Group B was cultured without ES from Day 0 to Day 3 and stimulated from Day 4 to Day 7. Neurons in Group A grew separately after 4 Day ES (Fig. 7A-Day 4). However, after 3 day culture without ES, the neurons in Group A aggregated again (Fig. 7A-Day 7), which indicated that cortical cells would form clusters after initial ES stopped. On the words, the effect of ES on cell morphology is a short-term effect. On the other hand, the aggregation appeared in Group B after 4 day culture without ES (Fig. 7B-Day 4), but the following 3 day ES did not disperse the neurons (Fig. 7B-Day 7), which indicated that the postponed ES was not enough to disperse the clustered neurons. Altogether, it indicated that ES could prevent clustering of cortical cells in the 3D-CNFs, but could not disperse already formed clusters of cortical cells.

**Fig. 8:**
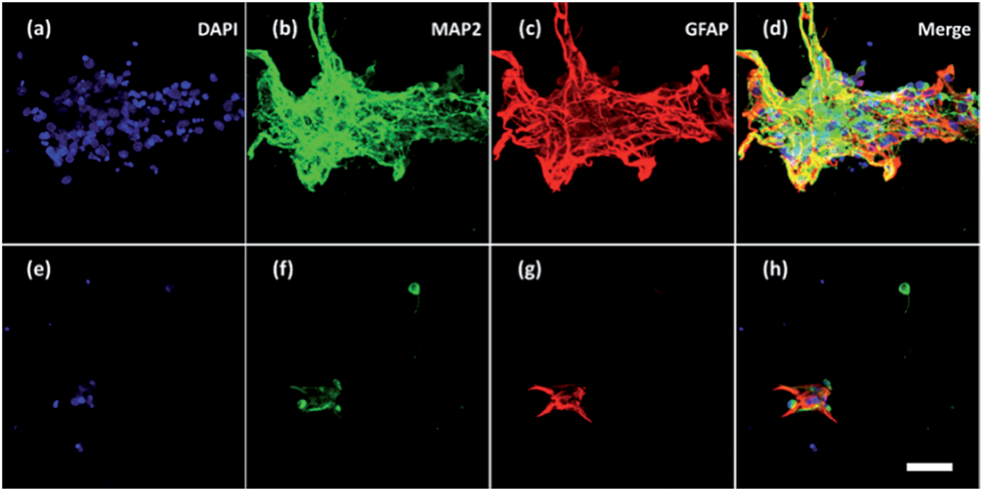
Confocal fluorescent images of neurons and glial cells on Day 7 in the 3D-CNFs (a–d) without and (e–h) with electrical stimulation. Blue: DAPI; green: MAP2; red: GFAP. Scale bar: 100 μm.

Furthermore, neurons and glial cells (mainly astrocytes) were stained by anti-MAP2 antibody and anti-GFAP antibody respectively. Neurons cultured without ES (Fig. 8a–d) aggregated while neurons cultured with ES (Fig. 8e–h) grew dispersedly. The MAP2 expression and GFAP expression indicated most of the neurons were growing closely with glial cells. It is known that glial cells could facilitate the migration and spread of neurons.^53^ With electrical stimulation, development of both neurons and glial cells could be promoted.^54,55^ For example, researchers have found that ES regulated astrocytes' proliferation and migration.^55^

**Fig. 9:**
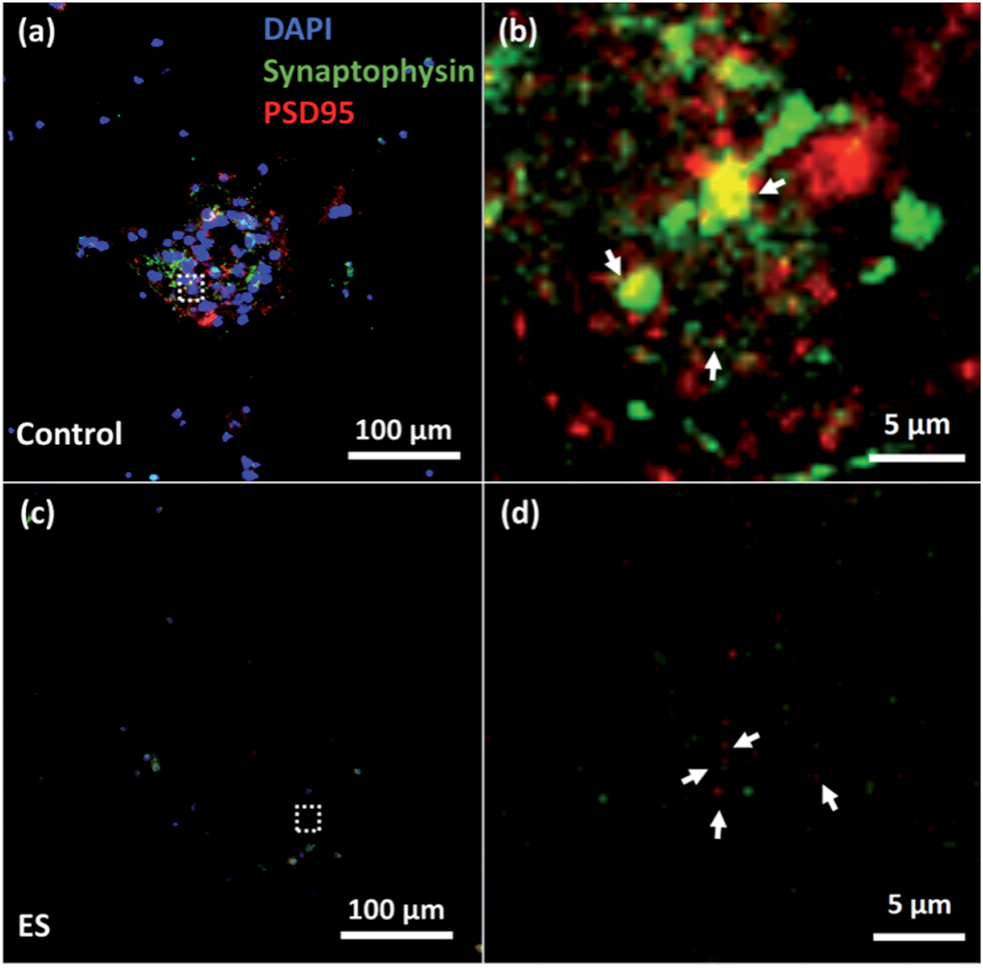
Immunostaining of synaptophysin and PSD 95 in 7 day cultured cortical cells in control (a and b) and ES (c and d) groups. (b) and (d) were regions of interest (indicated with dashed line) from (a) and (c) respectively. The synaptophysin was detected adjacent to PSD 95 (indicated with arrows).

To verify the formation of synapse, immunocytochemistry staining of both synaptophysin antibody (Life Technological, Singapore), a pre-synaptic biomarker, and post-synaptic density protein 95 (PSD95) (Millipore, Singapore), a post-synaptic biomarker, were utilized. The expression of synaptophysin and PSD95 of cells cultured for 7 days with or without ES were shown in Fig. 9. Synapses indicated by the adjacent or overlapped fluorescent spots of synaptophysin and PSD95 were found in both groups with and without ES.

Despite the mechanism how ES changed the clustering behavior of cells cultured in the 3D-CNFs is not completely clear, it had been reported that the clustering behavior could depend on the surface properties of the scaffolds, such as hydrophilicity,^53,56^ geometrical cues,^57^ as well as the electrical stimulation.^54,58^ In 2D substrates, it was reported that neuronal culture with electrical stimulation may increase neuron clusters,^54,58^ which was attributed to the heterogeneous conductivity of the substrate, *i.e.*, the conductivity in the different locations of the substrate was different. However, we found that electrical stimulation in 3D-CNFs prevented the cells from clustering, which could be attributed to the combined effects of the 3D structure and ES. Different from 2D substrates, the 3D structure provided a much larger space for cortical cells to spread and migrate in three-dimension (Fig. 5b), thus prevent the formation of clusters.

### 3.5 Effect of ES on proliferation of cortical cells

**Fig. 10:**
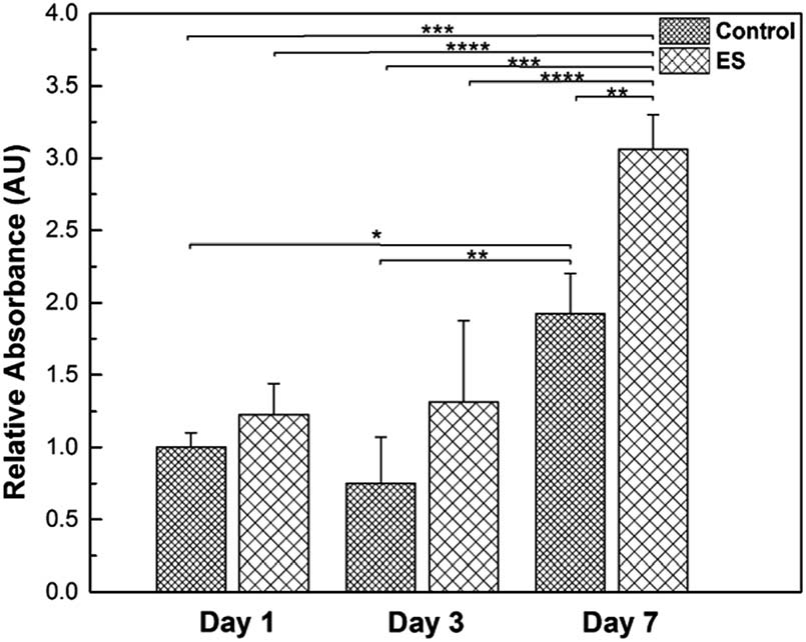
Proliferation (represented by relative absorbance in 570 nm) of cell cultures with or without electrical stimulation in Day 1, Day 3 and Day 7. * indicated *p* < 0.05, ** indicated *p* < 0.01, *** indicated *p* < 0.001 and **** indicated *p* < 0.0001. 3 wells for each group were measured.

Glial cells play important roles in the entire nervous system, such as providing physical supports for neurons and regulating the micro environment.^59^ To investigate the effects of ES to the glial cells, their proliferation in the 3D-CNFs was characterized while mature neurons do not proliferate.^60^ Results obtained from PrestoBlue kit were illustrated (Fig. 10) and the cell amounts were represented by relative absorbance. From Day 1 to Day 3, the means of cell amounts in ES groups were larger than that in control groups; but the increases were not statistically significant. In Day 7, the cell amounts showed a significant increase compared to Day 1 and Day 3 in both ES and control groups. Particularly, the cell amount in 3D-CNFs with ES was significantly larger than that without ES, which indicated a significant improvement on glial cell proliferation caused by ES through the 3D-CNFs. Two-way Analysis of Variance (ANOVA) test gave the main effect value for ES, *F* (2, 12) = 39.92, *p* < 0.0001 and the main effect value for culture time, *F* (2, 12) = 18.28, *p* = 0.0011, which indicated that electrical stimulation had even more significant influence than culture time on the proliferation of glial cells in 3D-CNFs.

The ES-related enhancement of cell proliferation is consistent with previous results obtained.^55,61–65^ The proliferation is probably attributed to the elevated calcium level because the voltage-gated calcium channels in the cell membrane could be activated by electrical stimulation.^65^ Furthermore, the surface properties of PPy coated nanofiber could be changed during electrical stimulation. For example, it had been reported that an increase of fibronectin absorbance on PPy with electrical stimulation.^66^ With the increase of protein absorbance on PPy surface, the surface would become more biocompatible and help cells spread, migrate and proliferate. For example, some astrocyte-secreted matricellular proteins are known to involve in cell proliferation, maturation and migration.^67^ Although the number of neurons is generally assumed to be a key determinant of brain function, glial cells have been valued as a major part of the brain.^59^ Regulation of glial cell proliferation could be an important tool in regeneration of specific part of the brain as the glia/neuron ratio varies along the brain.^68^

### 3.6 Effect of ES on maturation of neurons

**Fig. 11:**
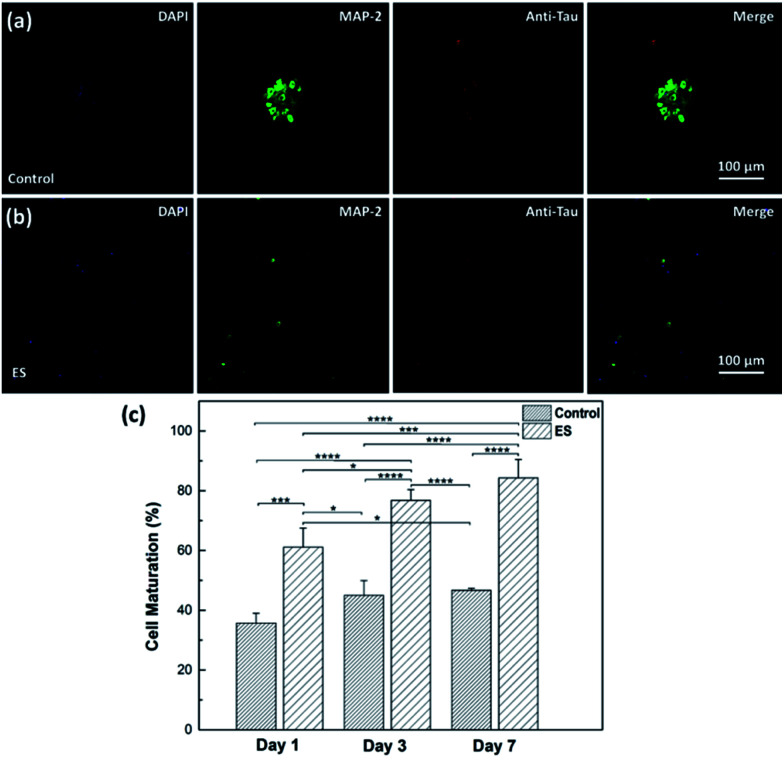
Fluorescent images of neurons in control (a) and ES (b) groups after 7 day culture and the maturation of neurons in 3D-CNFs (c) Day 1, Day 3 and Day 7. Green: MAP2; blue: DAPI; red: Tau. * indicated *p* < 0.05, ** indicated *p* < 0.01, *** indicated *p* < 0.001 and **** indicated *p* < 0.0001. 3 wells for each group on each day were measured.

The effect of ES on cortical cell cultures in 3D-CNFs was also characterized by neuron maturation, *i.e.*, the ratio of the number of mature neurons to the total number of neurons (Fig. 11). Anti-tau antibody staining was utilized to define maturation of neurons,^22^*i.e.*, mature neurons could be defined as neurons with tau expression. The total number of neurons was obtained by counting the cells with MAP2 expression in Imaris (Bitplane, USA). The single-channel and merged fluorescent images indicated the existing of mature neurons after 7 day culture in the control and ES group respectively (Fig. 11a and b). A significant enhancement of maturation happened in ES groups compared to control groups could be observed at every time point starting from Day 1 to Day 7. Without ES, the maturation in control groups reached 35.70% on Day 1, then slowly increased to 44.98% on Day 3 and finally reached 46.68% on Day 7. With ES, 61.07% of neurons in the 3D-CNFs were mature on Day 1 followed by a significant jump to 76.75% on Day 3, which increased to 84.27% on Day 7. From a two-way ANOVA, the main effect values were ES (*F* (2, 12) = 208.9, *p* < 0.0001) and culture time (*F* (2, 12) = 21.84, *p* = 0.0001), which suggested that both ES and culture time contributed significantly on the maturation of neurons in 3D-CNFs (Fig. 11c). The enhancement of neuron maturation may also be attributed to the signaling change, *i.e.*, the influx of Ca^2+^ induced by the depolarizing current, which can active the calmodulin-kinases to elicit neurite outgrowth and expedite neurites development.^22,26^

## 4. Conclusions

The biocompatibility of 3D PAN nanofibers with and without PPy coating had been demonstrated by culturing cortical cells. The fluffy 3D structure was shown to allow cells to penetrate into the interior space to form 3D cultures. Clustering behaviors of cultured cortical cells were different affected by both the coating and electrical stimulations. While smooth PAN 3D nanofibers showed dispersive cell distribution, PPy coated 3D-CNFs showed clusters of cortical cells. The proliferation data showed in Fig. 10 further indicated the biocompatibility of PPy-coated PAN scaffolds. The number of cells cultured in the 3D-CNFs was consistent from Day 1 to Day 3 without a significant decrease, and increased significantly from Day 3 to Day 7. Different from previous observations on 2D substrates, pulsed electrical stimulations could prevent formatting of cell clusters if applied at the beginning of culture, but could not disperse the clusters of cortical cells already formed. Furthermore, the electrical stimulations improved the proliferation of glial cells and accelerate neuron maturation. However, for the future clinical application, it might still has some concerns in the long-term use of PPy as a previous report mentioned the PPy-coated silicon tube has caused a light inflammation after half-year implantation in rats.^39^ A quantitative relation between of dose of implanted tissue and inflammation would be studied on the pre-clinical level.

In summary, the combined effects of the 3D conductive nanofibers and electrical stimulation on neurons and glial cells were investigated. This study enriched the growing body of evidence for using electrical stimulation and 3D conductive nanofibers to control the culture of cortical cells. The fluffy 3D nanofibrous structure and conductive properties provide a novel platform to explore a series of applications in neural tissue engineering.

## Conflicts of interest

There are no conflicts to declare.

## Supplementary Material
